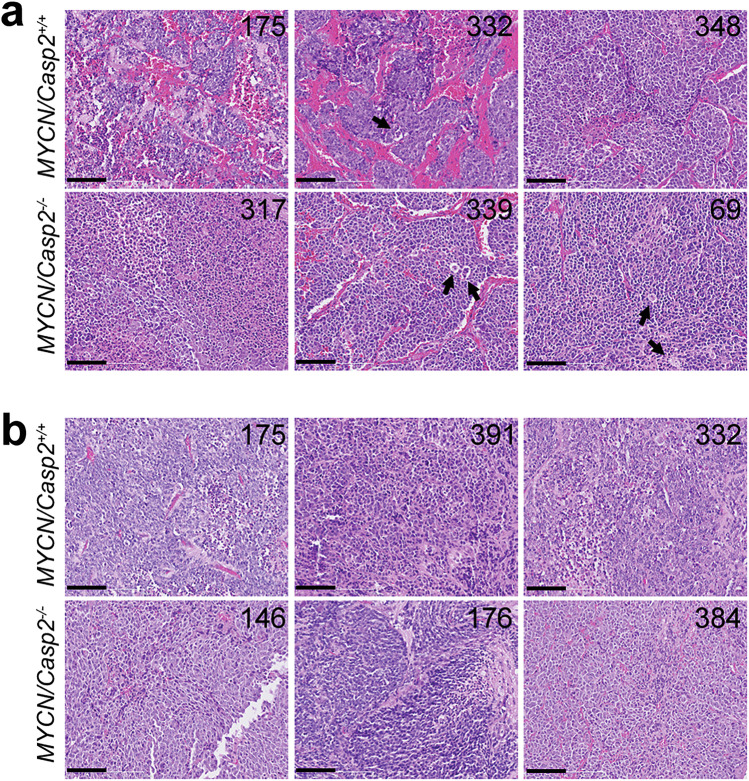# Correction: An unexpected role for caspase-2 in neuroblastoma

**DOI:** 10.1038/s41419-026-09023-2

**Published:** 2026-07-07

**Authors:** L. Dorstyn, J. Puccini, A. Nikolic, S. Shalini, C. H. Wilson, M. D. Norris, M. Haber, S. Kumar

**Affiliations:** 1 Centre for Cancer Biology, University of South Australia, Adelaide, SA 5001 Australia; 2 Department of Medicine, University of Adelaide, Adelaide, SA 5005 Australia; 3 Children’s Cancer Institute Australia for Medical Research, Lowy Cancer Research Centre, UNSW, Sydney, NSW 2052 Australia

Correction to: Cell Death Dis 2014;5:e1383. 10.1038/cddis.2014.342, published online 21 August 2014

Since the publication of this article, an error has been identified in Figure 2. In the originally published version of this manuscript, the thoracic tumor image from a *TH-MYCN/Casp2*^*+/+*^ mouse (#175) in Figure 2b, partially overlapped with the abdominal tumor image shown in Figure 2a for the same mouse. This incorrect image was inadvertently inserted during figure assembly. The figure has now been replaced with an appropriate, representative thoracic tumor image from mouse #175, sourced from the original image file.

Additional labelling errors in the same figure have also been corrected, including labelling of animal numbers and scale bar. The corrected figure and figure legend have been provided.

Importantly, these corrections do not change any data interpretation or the overall analysis of the tumour images or conclusions of the study. We apologise for these oversights and appreciate the opportunity to correct the record.

**Figure 2**. Histopathology of *TH-MYCN/Casp2*^+/+^ and *TH-MYCN/Casp2*^-/-^ neuroblastomas. Representative images (×40) of hematoxylin and eosin stained (a) abdominal tumors and (b) thoracic tumors, from mice of the indicated genotypes Three different tumor samples for each genotype are shown, with animal numbers indicated. Arrows highlight tangible body macrophages. Scale bar represents 100 µm.

**Figure 2 Original** 
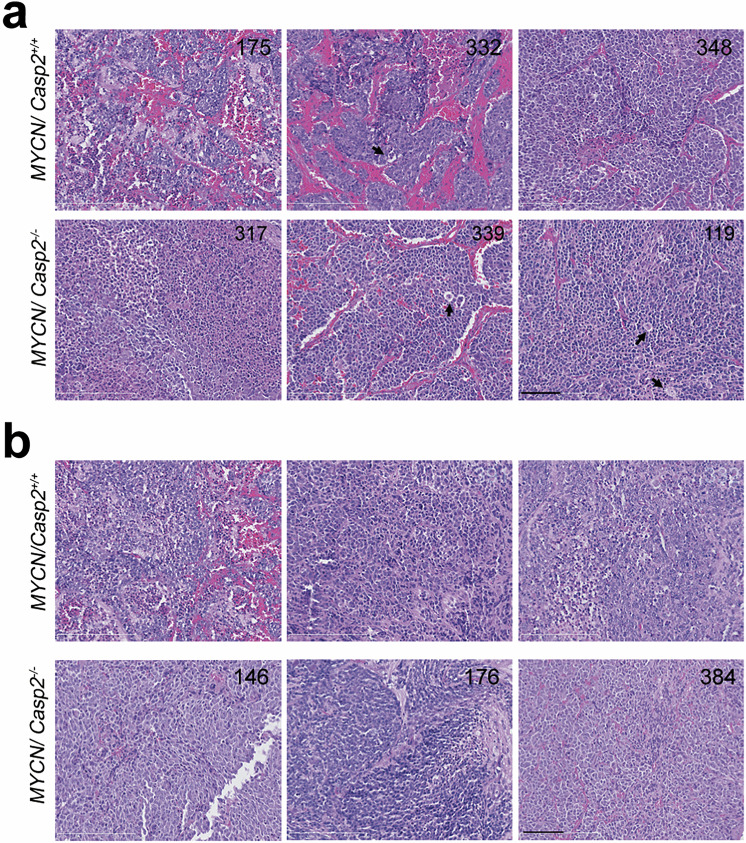


**Figure 2 Corrected**